# Supplementary material for: Kaempferia parviflora Rhizome Extract as Potential Anti-Acne Ingredient
Source: Molecules. 2022 Jul 8;27(14):4401. doi: 10.3390/molecules27144401 (PMC9321094; doi:10.3390/molecules27144401)
Supplement: Supplementary file 1 [file molecules-27-04401-s001.zip › molecules-1788418-supplementary.pdf]

molecules

Kaempferia parviflora Rhizome Extract as Potential Anti-Acne Ingredient

Pawee Sitthichai[1], Setinee Chanpirom [1,2], Tharakorn Maneerat [3,4,5], Rawiwan Charoensup[5,6], Thapakorn Tree-Udom[1,2], Punyawatt Pintathong[1], Surat Laphookhieo[3,4] and Tawanun Sripisut[1,2]*

[1] School of Cosmetic Science, Mae Fah Luang University, Chiang Rai 57100, Thailand; pawee2534@gmail.com (P.S.); setinee.cha@mfu.ac.th (S.C.); thapakorn.tre@mfu.ac.th (T.T.); punyawatt.pin@mfu.ac.th (P.P.)
[2] Phytocosmetics and Cosmeceuticals Research Group, Mae Fah Luang University, Chiang Rai 57100, Thailand
[3] School of Science, Mae Fah Luang University, Chiang Rai 57100, Thailand; wisanu.man@mfu.ac.th (T.M.); surat.lap@mfu.ac.th (S.L.)
[4] Center of Chemical Innovation for Sustainability (CIS), Mae Fah Luang University, Chiang Rai 57100, Thailand
[5] Medicinal Plants Innovation Center of Mae Fah Luang University, Mae Fah Luang University, Chiang Rai 57100, Thailand; rawiwan.cha@mfu.ac.th (R.C.)
[6] School of Integrative Medicine, Mae Fah Luang University, Chiang Rai 57100, Thailand
* Correspondence: tawanun.sri@mfu.a c.th; Tel.: +66-53-916-833

Table S1. Chemical profile of flavonoids in *K. parviflora* methanol extract.

Methanol extract				
**Peak number**	**Retention time (min)**	**Mass (g)**	**Chemical formula**	**Name of compounds**
1	16.45	312.0998	$C_{18}H_{16}O_5$	5,7,4′ -Trimethoxyflavone
2	17.08	312.0998	$C_{18}H_{16}O_5$	3,5,7-Trimethoxyflavone
3	17.70	298.0841	$C_{17}H_{14}O_5$	5-Hydroxy-7,4′-dimethoxyflavone
4	18.45	282.0892	$C_{17}H_{14}O_4$	5,7-Dimethoxyflavone
5	19.40	342.1103	$C_{19}H_{18}O_6$	3,5,7,4′-Tetramethoxyflavone
6	19.81	342.1104	$C_{19}H_{18}O_7$	5-Hydroxy-3,7,3′,4′-tetramethoxyflavone
7	19.94	268.0736	$C_{16}H_{12}O_4$	5-Hydroxy-7-methoxyflavone
8	20.14	298.0841	$C_{17}H_{14}O_5$	5-Hydroxy-3,7-dimethoxyflavone
9	20.74	328.0947	$C_{18}H_{16}O_6$	5-Hydroxy-3,7,4′-trimethoxyflavone

Table S2. Chemical profile of flavonoids in *K. parviflora* ethanol extract.

Ethanol extract				
**Peak number**	**Retention time (min)**	**Mass (g)**	**Chemical formula**	**Name of compounds**
1	16.35	312.0998	$C_{18}H_{16}O_5$	5,7,4′ -Trimethoxyflavone
2	17.08	312.0998	$C_{18}H_{16}O_5$	3,5,7-Trimethoxyflavone
3	17.73	298.0841	$C_{17}H_{14}O_5$	5-Hydroxy-7,4′-dimethoxyflavone

4	18.45	282.0892	$C_{17}H_{14}O_4$	5,7-Dimethoxyflavone
5	18.99	342.1103	$C_{19}H_{18}O_6$	3,5,7,4′-Tetramethoxyflavone
6	19.85	358.1052	$C_{19}H_{18}O_7$	5-Hydroxy-3,7,3′,4′-tetramethoxyflavone
7	19.94	268.0735	$C_{16}H_{12}O_4$	5-Hydroxy-7-methoxyflavone
8	20.15	298.0841	$C_{17}H_{14}O_5$	5-Hydroxy-3,7-dimethoxyflavone
9	20.77	328.0946	$C_{18}H_{16}O_6$	5-Hydroxy-3,7,4′-trimethoxyflavone

Table S3. Chemical profile of flavonoids in *K. parviflora* acetone extract.

**Acetone extract**				
**Peak number**	**Retention time (min)**	**Mass (g)**	**Chemical formula**	**Name of compounds**
1	16.35	312.0998	$C_{18}H_{16}O_5$	5,7,4′ -Trimethoxyflavone
2	17.04	312.0998	$C_{18}H_{16}O_5$	3,5,7-Trimethoxyflavone
3	17.69	298.0841	$C_{17}H_{14}O_5$	5-Hydroxy-7,4′-dimethoxyflavone
4	18.46	282.0892	$C_{17}H_{14}O_4$	5,7-Dimethoxyflavone
5	19.14	342.1103	$C_{19}H_{18}O_6$	3,5,7,4′-Tetramethoxyflavone
6	19.81	358.1052	$C_{19}H_{18}O_7$	5-Hydroxy-3,7,3′,4′-tetramethoxyflavone
7	19.94	268.0735	$C_{16}H_{12}O_4$	5-Hydroxy-7-methoxyflavone
8	20.14	298.0841	$C_{17}H_{14}O_5$	5-Hydroxy-3,7-dimethoxyflavone
9	20.77	328.0946	$C_{18}H_{16}O_6$	5-Hydroxy-3,7,4′-trimethoxyflavone

Table S4. Chemical profile of flavonoids in *K. parviflora* ethyl acetate extract.

**Ethyl acetate extract**				
**Peak number**	**Retention time (min)**	**Mass (g)**	**Chemical formula**	**Name of compounds**
1	16.35	312.0997	$C_{18}H_{16}O_5$	5,7,4′ -Trimethoxyflavone
2	17.08	312.0997	$C_{18}H_{16}O_5$	3,5,7-Trimethoxyflavone
4	18.45	282.0892	$C_{17}H_{14}O_4$	5,7-Dimethoxyflavone
5	18.99	342.1104	$C_{19}H_{18}O_6$	3,5,7,4′-Tetramethoxyflavone
6	19.85	358.1053	$C_{19}H_{18}O_7$	5-Hydroxy-3,7,3′,4′-tetramethoxyflavone
7	19.94	268.0735	$C_{16}H_{12}O_4$	5-Hydroxy-7-methoxyflavone
8	20.15	298.0841	$C_{17}H_{14}O_5$	5-Hydroxy-3,7-dimethoxyflavone
9	20.77	328.0946	$C_{18}H_{16}O_6$	5-Hydroxy-3,7,4′-trimethoxyflavone

Table S5. Chemical profile of flavonoids in *K. parviflora* dichloromethane extract.

Dichloromethane extract				
**Peak number**	**Retention time (min)**	**Mass (g)**	**Chemical formula**	**Name of compounds**
1	16.35	312.0997	$C_{18}H_{16}O_5$	5,7,4′ -Trimethoxyflavone
2	17.08	312.0997	$C_{18}H_{16}O_5$	3,5,7-Trimethoxyflavone
4	18.45	282.0892	$C_{17}H_{14}O_4$	5,7-Dimethoxyflavone
5	18.99	342.1103	$C_{19}H_{18}O_6$	3,5,7,4′-Tetramethoxyflavone
6	19.85	358.1052	$C_{19}H_{18}O_7$	5-Hydroxy-3,7,3′,4′-tetramethoxyflavone
8	20.15	298.0841	$C_{17}H_{14}O_5$	5-Hydroxy-3,7-dimethoxyflavone
9	20.77	328.0946	$C_{18}H_{16}O_6$	5-Hydroxy-3,7,4′-trimethoxyflavone

Table S6. Chemical profile of flavonoids in *K. parviflora n*-hexane extract.

n-Hexane extract				
**Peak number**	**Retention time (min)**	**Mass (g)**	**Chemical formula**	**Name of compounds**
4	18.39	282.0892	$C_{17}H_{14}O_4$	5,7-Dimethoxyflavone
5	19.46	342.1103	$C_{19}H_{18}O_6$	3,5,7,4′-Tetramethoxyflavone
6	19.88	358.1052	$C_{19}H_{18}O_7$	5-Hydroxy-3,7,3′,4′-tetramethoxyflavone
7	20.14	268.0735	$C_{16}H_{12}O_4$	5-Hydroxy-7-methoxyflavone
8	20.49	298.0841	$C_{17}H_{14}O_5$	5-Hydroxy-3,7-dimethoxyflavone
9	20.75	328.0946	$C_{18}H_{16}O_6$	5-Hydroxy-3,7,4′-trimethoxyflavone

Table S7. Viscosity profiles of gel-cream formulations.

**Conditions**	**Viscosity (cP)**			
	**Baseline**	**Week 4**	**Week 8**	**Week 12**
CB (AT)	7783.33 ± 76.37	8123.33 ± 142.24	7936.66 ± 40.41	8070.00 ± 117.89
CKP (AT)	7750.00 ± 105.83	8216.66 ± 98.14	7950.00 ± 70.02	8053.33 ± 96.09
CB (4 °C)	7783.33 ± 76.37	8330.00 ± 150.99	8030.00 ± 130.00	8206.66 ± 30.55
CKP (4 °C)	7750.00 ± 105.83	8193.33 ± 246.64	8103.33 ± 82.86	8073.33 ± 70.23

CB (45°C)	7783.33 ± 76.37	8363.33 ± 89.62	7923.33 ± 180.37	8053.333 ± 59.86
CKP (45 °C)	7750.00 ± 105.83	8503.33 ± 32.14	8166.333 ± 58.59	8050.00 ± 130.86

cP: centipoise; AT: ambient temperature storing condition; 4 °C: refrigerator storing condition; 45 °C: hot oven storing condition.

Table S8. pH profiles of gel-cream formulations.

Conditions	pH			
	Baseline	Week 4	Week 8	Week 12
CB (AT)	5.55 ± 0.00	5.40 ± 0.00	5.42 ± 0.02	5.43 ± 0.01
CKP (AT)	5.55 ± 0.01	5.39 ± 0.01	5.40 ± 0.01	5.39 ± 0.01
CB (4 °C)	5.55 ± 0.00	5.44 ± 0.01	5.48 ± 0.00	5.49 ± 0.01
CKP (4 °C)	5.55 ± 0.01	5.42 ± 0.00	5.46 ± 0.02	5.47 ± 0.01
CB (45 °C)	5.55 ± 0.00	5.40 ± 0.01	5.42 ± 0.01	5.48 ± 0.01
CKP (45 °C)	5.55 ± 0.01	5.39 ± 0.00	5.36 ± 0.00	5.39 ± 0.02

AT: ambient temperature storing condition; 4 °C: refrigerator storing condition; 45 °C: hot oven storing condition.

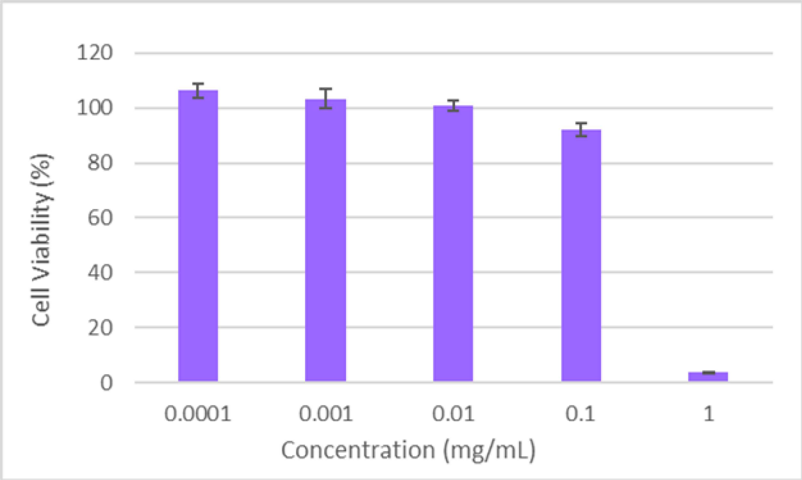

Figure S1. Human fibroblast cell viability with increasing concentrations of ethyl acetate extract of *K. parviflora*.

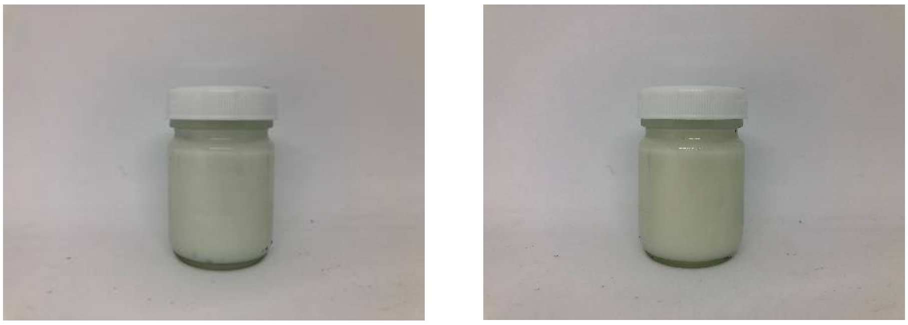

(**a**) (**b**)

Figure S2. Gel-cream base (**a**) and gel-cream containing ethyl acetate extract of *K. parviflora* (**b**).
